# Repair of Double‐Chamber Right Ventricle and Subvalvular Pulmonic Stenosis in a 78‐Year‐Old With Repaired Tetralogy of Fallot: Anesthetic Considerations and Review of the Literature

**DOI:** 10.1155/cria/8376547

**Published:** 2026-05-28

**Authors:** Robert Ricketts, Joseph Caruso, Erik Scott, Asma Habib, Jared P. Beller

**Affiliations:** ^1^ Department of Anesthesiology, Cincinnati Children’s Hospital Medical Center, 3333 Burnet Avenue, Cincinnati, Ohio, 45229, USA, cincinnatichildrens.org; ^2^ Department of Anesthesia & Perioperative Medicine, The Medical University of South Carolina, 167 Ashley Avenue, Charleston, South Carolina, 29425, USA, musc.edu; ^3^ Tallahassee Memorial Healthcare, Florida State University College of Medicine, 1300 Miccosukee Road, Tallahassee, Florida, 32308, USA, fsu.edu; ^4^ Department of Medicine, Division of Cardiovascular Medicine, University of Virginia, 200 Jeanette Lancaster Way, Charlottesville, Virginia, 22903, USA, virginia.edu; ^5^ Department of Surgery, Division of Cardiothoracic Surgery, University of Virginia, 200 Jeanette Lancaster Way, Charlottesville, Virginia, 22903, USA, virginia.edu

**Keywords:** adult congenital heart disease, anomalous coronary arteries, double-chamber right ventricle, right ventricular outflow tract obstruction, tetralogy of Fallot

## Abstract

**Background:**

Double‐chambered right ventricle (DCRV) is a rare congenital anomaly characterized by hypertrophied muscular bundles dividing the right ventricle into high‐ and low‐pressure chambers. It is frequently associated with congenital heart disease, particularly tetralogy of Fallot (TOF), but rarely presents in late adulthood.

**Case Presentation:**

We report the case of a 78‐year‐old man with a history of prior pulmonary valvotomy who presented with progressive dyspnea and was found to have severe subvalvular right ventricular outflow tract obstruction. Multimodal imaging and cardiac catheterization confirmed DCRV with near‐systemic right ventricular pressures and an anomalous left anterior descending coronary artery arising from the right coronary cusp. The patient underwent surgical resection of hypertrophied infundibular muscle bundles with patch augmentation of the main pulmonary artery. Intraoperative transesophageal echocardiography guided management and confirmed successful relief of obstruction. The patient had an uncomplicated postoperative course with resolution of symptoms.

**Discussion:**

This case highlights the late presentation of DCRV as a sequela of incompletely repaired TOF and underscores the importance of comprehensive imaging and careful surgical planning in the presence of anomalous coronary anatomy. Anesthetic management focused on maintaining right ventricular preload and coronary perfusion while avoiding dynamic right ventricular outflow tract obstruction.

**Conclusion:**

DCRV can present late in life in patients with prior congenital heart disease. Successful management requires a multidisciplinary approach with careful perioperative hemodynamic optimization.

## 1. Introduction

Tetralogy of Fallot (TOF) is a congenital heart lesion with significant heterogeneity in presentation and physiology. Most simply, TOF can present with minimal pulmonary valve (PV) stenosis and mimic the physiology of a large ventricular septal defect (VSD), or in its most severe form, it can present with complete atresia of the pulmonary artery leading to severe cyanosis and ductal‐dependent single ventricle physiology. However, more commonly, TOF can present as an anterior malalignment VSD with associated infundibular hypertrophy, pulmonary stenosis, and right ventricular outflow tract (RVOT) obstruction.

We report on a case of a 78‐year‐old individual with a history of a pulmonary valvotomy, a small residual perimembranous VSD, and infundibular hypertrophy causing subpulmonary stenosis, who likely had a less complex incompletely palliated TOF lesion. The patient presented for repair of a double‐chambered right ventricle (DCRV) and critical subvalvular pulmonary stenosis.

## 2. Case Presentation

**FIGURE 1:**
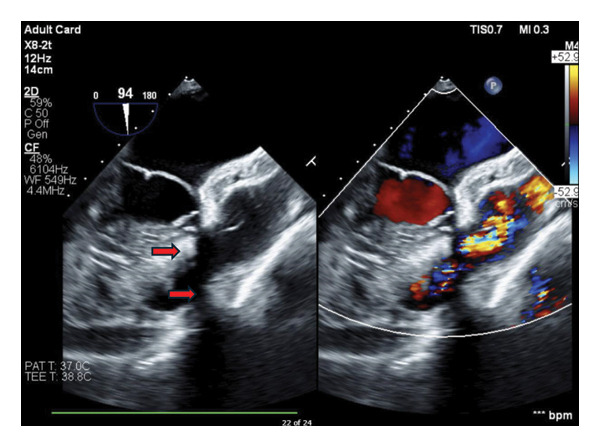
View of the RVOT on TEE demonstrating subvalvular RVOT muscle bands (marked with red arrows) and flow acceleration on color Doppler.

A 78‐year‐old man with a reported history of congenital PV stenosis, main pulmonary artery (MPA) hypoplasia, and a VSD presented to our adult congenital cardiology clinic with progressive shortness of breath and dyspnea on exertion. His past medical history was significant for chronic obstructive pulmonary disease, tobacco smoking, and past PV surgery. According to the patient, he had undergone valvulotomy of his PV in 1960 at the age of 15. Given the year of his repair, no historical surgical records were found. Preoperative transthoracic (TTE) and transesophageal echocardiography (TEE) demonstrated normal PV structure and function but noted subvalvular flow acceleration with a mean systolic gradient of 61 mmHg and peak systolic gradient of 100 mmHg across the RVOT (Figure 1) due to infundibular muscle bundles. Additionally, a small residual perimembranous VSD was noted. A CT heart with contrast confirmed this severe subpulmonary stenosis but also noted that the patient’s left anterior descending (LAD) coronary artery originated from the right coronary sinus and traversed across the RVOT.

The patient was referred for preoperative cardiac catheterization. Ventricular angiography was performed, demonstrating right ventricular morphology as two separate RV chamber pressures separated by a bundle of hypertrophied infundibular muscle in the RVOT. The superior chamber of the right ventricle, immediately proximal to the PV, demonstrated a pressure of 25/10 mmHg. Pressure in the inferior chamber of the right ventricle, immediately distal to the tricuspid valve, measured 95/15 mmHg. MPA pressures and pulmonary vascular resistance were normal. The patient had a normal systolic blood pressure. With a finding of a near‐systemic right ventricular pressure in the inferior portion of the RV, the patient was diagnosed with a DCRV. Coronary angiography showed a right dominant system, demonstrated the left circumflex coronary artery originating from the left coronary cusp and an absent left main coronary artery, and confirmed the LAD originating from the right coronary cusp. The right coronary artery originated from a separate and distinct ostium off the right coronary cusp.

Given the patient’s worsening symptoms, the decision was made to proceed with operative repair. The surgical plan was to resect the infundibular muscle bundles that were generating the severe subvalvular stenosis in the RVOT. Given the course of the anomalous LAD, infundibular RVOT patch repair was not a viable surgical option.

The patient was taken to the operating room, and a preinduction right radial arterial line was placed. An additional large bore IV was placed, and anesthesia was induced via intravenous fentanyl, ketamine, and rocuronium. The patient was intubated with an 8.0 cuffed ETT. Since the operation was going to be right sided and involve the RVOT, a Swan‐Ganz catheter was not considered for this patient. As such, a 7 Fr triple‐lumen CVC was placed in the patient’s right internal jugular vein for central venous access. An additional large‐bore IV was secured for volume administration. Anesthesia was maintained using ketamine, isoflurane, and fentanyl. Isotonic crystalloid fluids, phenylephrine, and norepinephrine (NE) were administered prior to cardiopulmonary bypass (CPB) to maintain RV perfusion given the patient’s known RV systolic pressures. IV methadone and tranexamic acid bolus with subsequent infusion were administered per our institutional adult cardiac anesthesia protocols.

Repeat sternotomy and cannulation were uneventful. The patient maintained his blood pressure well, only requiring a low‐dose background NE infusion and ongoing fluid administration. Intraoperative TEE was used to assess RV filling and contractility with the goal of optimizing right ventricular filling and avoiding hypovolemia or hyperdynamic function. Central bicaval cannulation was performed, and CPB was initiated. The heart was arrested via aortic cross‐clamping and the administration of cold anterograde Del Nido cardioplegia solution.

While on bypass, the surgical team began their repair by opening the right atrium and examining the right ventricle across the tricuspid valve. Muscle bundles in this region were divided and excised with care not to disturb the subvalvular apparatus of the tricuspid valve. The dissection and resection of muscle bundles were carried toward the RVOT. The PV was visualized from the right ventricle confirming a normal appearance and appropriate size. The tricuspid valve was tested and shown to be competent. The patient was rewarmed, and the right atrium was closed.

After de‐airing of the heart, the heart was restarted by removing the cross‐clamp and initiation of a low‐dose epinephrine infusion of 2 mcg/min. The heart was refilled and allowed to eject while being closely monitored from the field and by TEE to assess for dynamic RVOT obstruction or persistent RV obstruction from the infundibular muscle band. Intraoperative TEE demonstrated considerable improvement of the RVOT turbulence; however, there still was a muscle band in the infundibulum that was significantly contributing to flow acceleration across the RVOT. The decision was made to rearrest the heart, go back on CPB, and address this band.

After the aorta was cross‐clamped again and administration of cold Del Nido solution provided arrest, the MPA was opened longitudinally. Additional hypertrophied muscle was identified on the RV free wall and septal wall just under the PV that had not been visualized through the initial tricuspid valve approach. After further RVOT resection, the decision was made to close the MPA using a bovine pericardial patch, simultaneously augmenting the mildly hypoplastic MPA.

**FIGURE 2:**
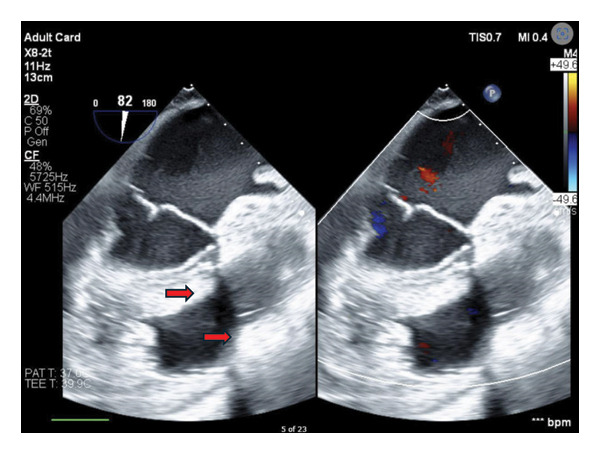
View of the RVOT on TEE demonstrating relief of subvalvular RVOT muscle bands (marked with red arrows) and resolution of flow acceleration on color Doppler.

The patient was again warmed, and the heart was de‐aired and restarted by removing the aortic cross‐clamp and restarting our epinephrine infusion. Once the heart was filled, intraoperative TEE demonstrated resolution of the RVOT obstruction with a decrease in velocity across the subpulmonary region, good biventricular function, and no obvious issues or injury to the tricuspid or PVs (Figure 2). The patient was successfully weaned from CPB. Heparin was reversed with the administration of protamine. The patient did well during his time on CPB, only requiring a NE infusion and intermittent vasopressin infusion to maintain adequate blood pressures.

Post‐CPB, pump‐blood was gently returned to the patient. The patient further received 1 unit of cryoprecipitate and 1 unit of FFP post protamine administration as guided by viscoelastic rewarming labs performed on the Quantra QPlus analyzer and our institutional goal‐directed transfusion protocols. Post‐repair hemodynamic management was again focused on maintaining adequate RV preload and avoiding RV hyperdynamic states. The patient arrived to our CVICU intubated on low‐dose epinephrine and NE infusions but ultimately hemodynamically stable.

**FIGURE 3:**
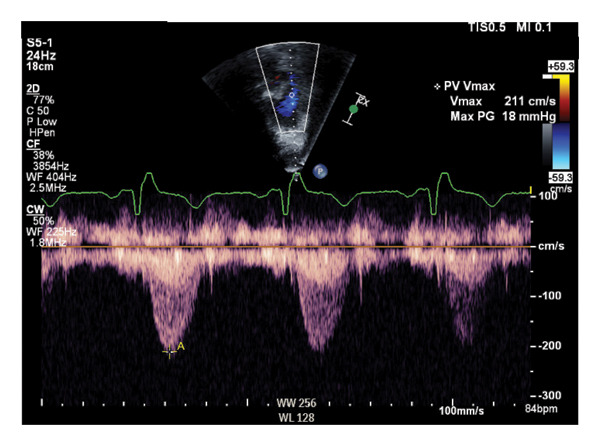
Continuous wave Doppler on TTE demonstrating a peak systolic gradient of 18 mm Hg across RVOT.

The patient went on to have an uneventful recovery. He was extubated and weaned off all vasopressor and ionotropic support by postoperative day (POD) 2. TTE on POD 6 showed normal subpulmonary velocities with a peak systolic pressure gradient of approximately 18 mmHg across the RVOT (Figure 3). The patient was discharged home on POD 8. He had full resolution of his symptoms and significant functional improvement on outpatient cardiology follow‐up.

## 3. Discussion

TOF is a heterogeneous congenital cardiac lesion with a wide range of anatomic and physiologic presentations. In patients who undergo incomplete or palliative repair, progressive RVOT obstruction may develop over time. In this case, prior pulmonary valvotomy without complete relief of subvalvular obstruction likely permitted progressive hypertrophy of infundibular muscle bundles, ultimately resulting in DCRV and symptomatic right ventricular hypertension decades later.

DCRV is a rare condition seen in 0.5%–2.0% of congenital heart disease [1]. It derives its name from the appearance of the RV on cardiac catheterization angiography demonstrating two separate ventricular “chambers” or portions to the right ventricle separated by the thickened muscle bundles in the RVOT with mid‐cavitary obstruction. This subpulmonary hypertrophy effectively divides the right ventricle into two distinct chambers of pressure. There is a low‐pressure chamber located more distally or closer to the PV, and a higher‐pressure chamber located more proximally or closer to the tricuspid valve [2]. Most cases of DCRV present in childhood, but more mild and progressive obstruction can present in adults [3]. DCRV rarely occurs in isolation and is often found with concurrent structural congenital heart disease. It has been associated with lesions like subaortic stenosis, TOF, double‐outlet RV, anomalous pulmonary venous return, and transposition of the great vessels, among others. However, the strongest associated lesion is a perimembranous VSD, which is seen in 63%–90% of DCRV [3–5]. It is theorized that the high‐pressure and high‐velocity flow across the small VSD hits the tissue in the RVOT, leading to adaptive hypertrophy over time, which generates the DCRV morphology. As DCRV is so strongly associated with CHD, some have argued that the entity should be viewed as an extreme manifestation of other CHD and not so much a discrete clinical entity in and of itself [1]. As such, the observation of a DCRV should prompt a thorough workup and search for other congenital heart disease lesions.

The clinical diagnosis of DCRV can be challenging and may contribute to adult patients presenting late in life. Presenting symptoms such as dyspnea, angina, and syncope often mimic more common cardiac pathology such as myocardial ischemia and or arrhythmia [6–8]. Furthermore, TTE, a standard first‐pass test for cardiologists, does not do well in diagnosing DCRV with some case series showing TTE to be diagnostic only in 15% of adult patients with the condition [5, 9]. This is likely secondary to the location of the stenosis, creating difficulty in lining up a spectral Doppler signal parallel to the flow between chambers. TEE, cardiac catheterization, cardiac CT, and cardiac MRI are superior to TTE at diagnosing DCRV [1]. Specifically, in adults, TEE has been shown to diagnose DCRV 100% of the time in some case series due to better visualization of RV structures and better alignment of Doppler interrogation of the two chambers [5]. TTE was not able to diagnose our patient’s DCRV. His initial TTE identified his subvalvular pulmonary stenosis and turbulent flow, but his dual pressures and pressure gradients observed in the RV were better identified and delineated on subsequent TEE and cardiac catheterization, including right ventricular angiography. Our patient’s development of DCRV was likely due to already present subpulmonary infundibular muscle bundles and subsequent hypertrophy caused by the left‐to‐right high‐pressure shunt across his small VSD.

Optimal treatment of DCRV is felt to be surgical removal of the hypertrophic tissue. While there is no consensus indication for repair of DCRV, some large case series have cited pressure gradients greater than 40 mm Hg between the RV chamber and MPA and or symptoms of heart failure as reasons to repair [2]. Our patient had symptoms of heart failure and a mean RVOT gradient exceeding 60 mm Hg, which was the primary indication for his repair. When DCRV has been repaired with lower pressure gradients or the absence of heart failure symptoms, patients generally had concurrent congenital heart lesions that were the primary indication of repair [2, 10]. Such lesions include VSDs, VSDs with pulmonary overcirculation, VSDs causing aortic valve insufficiency, and valvular pulmonic stenosis. Typically, repair of the DCRV is accomplished via right atriotomy; however, there are reports of repair of DCRV being done via right ventriculotomy and or pulmonary trunk incision [2, 10–12]. Resection of the causative muscle band has many potential complications. Papillary muscles can originate near this band of muscle, especially if the muscle band is hypertrophic change in the crista supraventricularis [1]. Repair of DCRV has been shown to occasionally cause incompetence of the tricuspid valve by disruption of the subvalvular apparatus [10, 11]. Fortunately, our patient’s tricuspid valve showed no signs of compromise post‐repair. Another common complication seen in repair of the DCRV is disruption of the subatrioventricular nodal conduction system. The right atrioventricular bundle runs along the septomarginal trabecula and can be damaged when muscular resection is performed. The moderator band itself can contribute to the obstruction causing DCRV. Surgical resection performed on either of these structures can generate a right bundle block. Indeed, some case series have reported rates upwards of 46% of postoperative right bundle block [11].

Another important consideration for patients with suspected TOF requiring cardiac surgery is the presence of anomalous coronary arteries (ACAs). While there have been varying reports in the literature of the prevalence of ACAs in TOF, a recent large meta‐analysis by Koppel et al. concluded that ACAs are seen in 5%–7% of TOF patients, with the most common association being the LAD originating from the right coronary cusp and crossing the RVOT [13]. Anomalous coronaries can have a significant impact on surgical technique and approach during repair and require diligent preoperative identification and planning. Cardiac catheterization and cardiac CTA are commonly performed in TOF patients to delineate coronary anatomy. If an ACA crosses the RVOT, this complicates intervention on the RVOT by prohibiting patch augmentation. It can also be prohibitive in cardiac catheterization transcatheter PV replacement. Intraoperatively, the LAD originating off the right coronary cusp makes de‐airing of the heart especially important, as air emboli can affect either or both ventricles.

These anatomic and physiologic considerations have important implications for perioperative anesthetic management. The anesthetic management of patients with DCRV and severe RVOT obstruction is centered on preservation of RV function, maintenance of coronary perfusion, and prevention of hemodynamic conditions that may exacerbate RVOT obstruction. In this patient, near‐systemic right ventricular pressures rendered RV perfusion highly dependent on adequate systemic vascular resistance (SVR) and diastolic pressure [9, 14, 15].

The induction strategy emphasized hemodynamic stability and avoidance of decreases in SVR. Ketamine was selected as the primary induction agent due to its sympathomimetic properties, which help maintain SVR and support coronary perfusion. Fentanyl was used to blunt sympathetic responses while minimizing myocardial depression. Rocuronium facilitated controlled airway management without significant hemodynamic effects. Agents such as propofol, which can cause vasodilation and reduce SVR, were avoided or used cautiously.

Hemodynamic goals during induction and maintenance included preservation of right ventricular preload, maintenance of SVR to support coronary perfusion, avoidance of tachycardia to preserve diastolic filling, and prevention of hyperdynamic states that could exacerbate dynamic RVOT obstruction. Maintenance of sinus rhythm and atrioventricular synchrony was also prioritized.

Monitoring considerations included placement of a preinduction arterial line for continuous blood pressure monitoring and central venous access for volume resuscitation and vasoactive medications. Intraoperative TEE was essential for real‐time assessment of RV size, contractility, preload, and detection of residual or dynamic RVOT obstruction, particularly during separation from CPB.

Particular attention was paid to mean arterial pressure as a surrogate for right ventricular coronary perfusion, central venous pressure trends to guide preload optimization, and TEE‐derived assessment of right ventricular size, contractility, preload status, and outflow tract gradients. These parameters were critical in distinguishing hypovolemia, right ventricular dysfunction, and dynamic RVOT obstruction.

Vasoactive medications should be immediately available and selected based on physiologic goals. NE serves as the primary agent to maintain SVR and coronary perfusion, while phenylephrine can be used for rapid augmentation of vascular tone without increasing heart rate. Epinephrine may be required for inotropic support in the setting of right ventricular dysfunction, and vasopressin can be used as an adjunct in vasoplegic states with minimal impact on pulmonary vascular resistance.

Emergency considerations in this type of physiology also include the risk of dynamic RVOT obstruction following relief of fixed obstruction. This condition is characterized by a hyperdynamic, underfilled right ventricle with reduced cardiac output and may mimic right ventricular failure [15]. As such, real‐time echocardiography is critical in distinguishing between these two entities, especially after chest closure. Management includes volume resuscitation, reduction of inotropic support, maintenance of SVR, and echocardiographic confirmation of diagnosis [15]. Given this patient’s long‐standing severe RVOT obstruction, we closely monitored him for a post repair dynamic RVOT obstruction. Prevention of dynamic RVOT obstruction relies on vigilant monitoring, maintenance of adequate RV preload, and judicious use of inotropic agents in the post‐CPB and postoperative setting.

## 4. Conclusion

This case illustrates the rare late presentation of DCRV in an elderly patient with a history of incompletely repaired TOF, highlighting the long‐term sequelae of earlier surgical strategies. As advances in diagnosis and management have increased survival into adulthood for patients with congenital heart disease, clinicians are increasingly encountering complex, late manifestations of prior repairs. This underscores the importance of maintaining a high index of suspicion for residual or progressive RVOT obstruction and recognizing the wide variability in presentation among this growing population. Multimodal imaging, particularly TEE and cardiac catheterization, remains essential for accurate diagnosis and operative planning, especially in the presence of complex anatomy such as ACAs. From an anesthetic perspective, meticulous attention to right ventricular preload, coronary perfusion, and avoidance of dynamic RVOT obstruction is critical to optimize perioperative outcomes. Ultimately, this case highlights both the evolving nature of congenital heart disease across the lifespan and the importance of a multidisciplinary approach tailored to the unique physiology of adult congenital patients undergoing cardiac surgery.

## Author Contributions

Robert Ricketts: conceptualization, writing–original draft, clinical management, and manuscript editing.

Joseph Caruso: writing–review and editing.

Erik Scott: surgical management and manuscript review.

Asma Habib: cardiology evaluation and manuscript review.

Jared P. Beller: surgical management, supervision, and manuscript review.

## Funding

This research received no external funding.

## Ethics Statement

Ethical approval was not required for this case report in accordance with institutional policies.

## Consent

Written informed consent was obtained from the patient for publication of this case report and any accompanying images. A copy of the written consent is available for review by the Editor‐in‐Chief of this journal upon request.

## Conflicts of Interest

The authors declare no conflicts of interest.

## Data Availability

No datasets were generated or analyzed during the preperation of this manuscript.
